# Genome-wide association study of adipocyte lipolysis in the GENetics of adipocyte lipolysis (GENiAL) cohort

**DOI:** 10.1016/j.molmet.2020.01.009

**Published:** 2020-01-25

**Authors:** Agné Kulyté, Veroniqa Lundbäck, Cecilia M. Lindgren, Jian'an Luan, Luca A. Lotta, Claudia Langenberg, Peter Arner, Rona J. Strawbridge, Ingrid Dahlman

**Affiliations:** 1 Lipid laboratory, Department of Medicine Huddinge, Karolinska Institute, Stockholm, Sweden; 2 Big Data Institute at the Li Ka Shing Center for Health Information and Discovery, University of Oxford, Oxford, UK; 3 Wellcome Center for Human Genetics, Nuffield Department of Medicine, University of Oxford, Oxford, UK; 4 National Institute for Health Research Oxford Biomedical Research Center, Oxford University Hospitals NHS Foundation Trust, John Radcliffe Hospital, Oxford, UK; 5 Program in Medical and Population Genetics, Broad Institute, Cambridge, MA, USA; 6 MRC Epidemiology Unit, University of Cambridge, Cambridge, UK; 7 Institute of Health and Wellbeing, University of Glasgow, Glasgow, UK; 8 Department of Medicine Solna, Karolinska Institute, Stockholm, Sweden; 9 Health Data Research UK, UK

**Keywords:** Genetic variants, Lipolysis, Subcutaneous, Adipocytes, Gene expression

## Abstract

**Objectives:**

Lipolysis, hydrolysis of triglycerides to fatty acids in adipocytes, is tightly regulated, poorly understood, and, if perturbed, can lead to metabolic diseases including obesity and type 2 diabetes. The goal of this study was to identify the genetic regulators of lipolysis and elucidate their molecular mechanisms.

**Methods:**

Adipocytes from abdominal subcutaneous adipose tissue biopsies were isolated and were incubated without (spontaneous lipolysis) or with a catecholamine (stimulated lipolysis) to analyze lipolysis. DNA was extracted and genome-wide genotyping and imputation conducted. After quality control, 939 samples with genetic and lipolysis data were available. Genome-wide association studies of spontaneous and stimulated lipolysis were conducted. Subsequent *in vitro* gene expression analyses were used to identify candidate genes and explore their regulation of adipose tissue biology.

**Results:**

One locus on chromosome 19 demonstrated genome-wide significance with spontaneous lipolysis. 60 loci showed suggestive associations with spontaneous or stimulated lipolysis, of which many influenced both traits. In the chromosome 19 locus, only *HIF3A* was expressed in the adipocytes and displayed genotype-dependent gene expression. *HIF3A* knockdown *in vitro* increased lipolysis and the expression of key lipolysis-regulating genes.

**Conclusions:**

In conclusion, we identified a genetic regulator of spontaneous lipolysis and provided evidence of *HIF3A* as a novel key regulator of lipolysis in subcutaneous adipocytes as the mechanism through which the locus influences adipose tissue biology.

## Highlights

• A genome-wide association study identified a chromosome 19 locus that was associated with lipolysis in adipose tissue.
• Of the genes in the locus, only *HIF3A* was strongly expressed during adipocyte differentiation.
• *In vitro* analyses demonstrated that *HIF3A* plays a key role in the regulation of adipocyte lipolysis.

## 1 Introduction

Release of fatty acids through hydrolysis (lipolysis) of triglycerides in fat cells plays a major role in energy homeostasis. Lipolysis is highly regulated in a species-specific fashion. In humans, only catecholamines and natriuretic peptides have a strong lipolytic action among hormones as reviewed [[1], [2], [3]]. Human fat cells also have spontaneous lipolytic activity [1]. Inter-individual variations in lipolysis have important clinical impact. Large cross-sectional studies show significant relationships between high spontaneous or low catecholamine-stimulated lipolysis and a variety of metabolic conditions such as obesity and insulin resistance [[4], [5], [6]]. A recent prospective study demonstrated that subjects with high spontaneous lipolysis activity and low catecholamine-stimulated lipolysis had a markedly increased risk of future development of excess body fat, insulin resistance, and type 2 diabetes/glucose intolerance [7].

There is a strong genetic component to clinical traits of relevance to lipolysis, including body fat (measured by body mass index, BMI) [8], the location of stored lipids (as measured by waist-to-hip ratio (WHR) adjusted for BMI, WHRadjBMI) [9], and circulating triglyceride levels [10]. It is therefore likely that lipolysis is also regulated, at least in part, by genetic variations. Indeed, studies of monozygotic twins demonstrated a strong within-pair resemblance of spontaneous and catecholamine-stimulated lipolysis [11,12] and we reported that family history of type 2 diabetes is associated with increased spontaneous adipocyte lipolysis [13]. A recent large study on expression quantitative trait loci (eQTLs) in subcutaneous adipose tissue (SAT), the largest fat depot in the body, strongly suggests a genetic regulation of fat cell function [14].

To shed new light on the genetic influence on lipolysis, we conducted a genome-wide association study (GWAS) on 939 subjects from the GENiAL (GENetics of Adipocyte Lipolysis) cohort with phenotyping of spontaneous and catecholamine-stimulated lipolysis in SAT.

## 2 Materials and methods

### 2.1 Participants

The GENiAL cohort includes 273 men and 718 women whose characteristics are presented in Table 1. The participants were recruited between 1986 and 2016 by local advertisements for studies to examine the regulation of fat cell function with a focus on lipolysis. The recruitment period was extensive due to the labor-intensive methodology used for adipocyte analysis (only a few samples a week are possible); however, a single laboratory and consistent selection and analysis protocols were employed throughout.

**Table 1:** Demographics of GENiAL cohort.

	Men			Women		
	nonobese	obese	*P*	nonobese	obese	*P*
lean/obese (n)	177	96		269	449	
age (years)	46(15)	43(12)	0.049	40(13)	41(10	0.4
height (cm)	178(10)	181(7)	0.0089	167(6)	166(6)	0.0038
body weight (kg)	81(10)	125(18)	<0.0001	67(9)	108(17)	<0.0001
BMI (kg/m^2^)	25(2)	38(5)	<0.0001	24(3)	39(5)	<0.0001
waist (cm)	92(9)	126(13)	<0.0001	84(10)	119(13)	<0.0001
WHR	0.95(0.06)	1.05(0.05)	<0.0001	0.86(0.07)	0.96(0.07)	<0.0001
systolic blood pressure (mm Hg)	129(15)	140(18)	<0.0001	120(16)	130(16)	<0.0001
diastolic blood pressure (mm Hg)	78(10)	85(12)	<0.0001	74(9)	79(10)	<0.0001
plasma total cholesterol (mmol/l)	5.1(1.3)	5.3(1.4)	0.33	4.9(1.1)	5.0(1.0)	0.15
plasma HDL cholesterol (mmol/l)	1.2(0.4)	1.0(0.2)	<0.0001	1.6(0.4)	1.2(0.3)	<0.0001
plasma triacylglycerides (mmol/l)	1.70(1.98)	2.33(2.49)	0.038	1.06(0.64)	1.52(0.86)	<0.0001
plasma nonesterified fatty acids (mmol/l)	0.49(0.17)	0.62(0.21)	<0.0001	0.62(0.21)	0.72(0.24)	<0.0001
plasma glycerol (μmol/l)	59.9(27.2)	78.4(29.9)	<0.0001	81.1(42.6)	116.9(54.1)	<0.0001
fasting plasma glucose (mmol/l)	5.4(1.2)	6.3(2.3)	0.0005	5.0(0.7)	5.6(1.4)	<0.0001
fasting serum insulin (mU/l)	7.8(5.2)	20.5(11.8)	<0.0001	6.2(3.3)	14.7(7.9)	<0.0001
HOMA-IR	2.01(1.95)	6.02(4.31)	<0.0001	1.40(0.87)	3.82(2.88)	<0.0001
Cell volume (pl)	483(157)	821(193)	<0.0001	473(177)	860(180)	<0.0001
Spontaneous lipolysis (mmol glycerol/2hrs/ESAT)	0.8(0.7)	3.8(2.8)	<0.0001	1.0(0.9)	3.3(2.9)	<0.0001
Stimulated lipolysis (mmol glycerol/2hrs/ESAT)	6.6(5.2)	3.6(1.6)	<0.0001	9.1(6.4)	5.8(7.0)	<0.0001

Where: obese is defined as BMI>30 kg/m^2^; Spontaneous lipolysis rate was calculated as glycerol release divided by the lipid weight of the incubated fat cells; Stimulated lipolysis was calculated as the quotient of glycerol release at the maximum effective isoprenaline concentration divided by the spontaneous rate (no hormone present) of glycerol release from the isolated fat cells. continuous variables are presented as mean (sd), groups were compared with Student's t-test (unpaired). ESAT is estimated amount of abdominal subcutaneous adipose tissue corresponding to the region for needle biopsy.

The GENiAL cohort was used to analyze multiple adipose phenotypes, but all of the participants in this study had undergone baseline examination in our laboratory and their lipolysis and DNA measurements were available (Supplementary Fig. 1). A total of 939 study subjects with adipocyte lipolysis measurements were included. Descriptions of the study subjects are presented in Table 2. Overall, 57% were obese (defined as BMI ≥ 30 kg/m^2^). All lived in Stockholm, Sweden. A total of 194 of the participants had type 2 diabetes, hypertension, or dyslipidemia alone or in different combinations. None were treated with insulin, glitazones, or glucagon-like peptide analogues. Data on clinical variables and rates of fat cell lipolysis were published previously [5,6]. The current study was approved by the local committee on ethics and explained in detail to each subject. Informed consent was obtained from all of the participants; this was in written form since 1996.

**Table 2:** Descriptions of the GENiAL participants included in this study.

	Men			Women		
	nonobese	obese	*P*	nonobese	obese	*P*
lean/obese (n)	89	163		245	442	
age (years)	47(15)	43(13)	0.044	40(13)	41(10)	0.7
height (cm)	178(6)	181(7)	0.0049	167(6)	166(6)	0.0076
body weight (kg)	80(10)	125(18)	<0.0001	67(9)	108(17)	<0.0001
BMI (kg/m^2^)	25(2)	38(5)	<0.0001	24(3)	39(5)	<0.0001
waist (cm)	92(9)	126(13)	<0.0001	84(10)	119(13)	<0.0001
WHR	0.95(0.06)	1.05(0.05)	<0.0001	0.86(0.07)	0.96(0.07)	<0.0001
systolic blood pressure (mm Hg)	129(15)	140(19)	<0.0001	120(16)	130(16)	<0.0001
diastolic blood pressure (mm Hg)	79(10)	85(12)	<0.0001	74(10)	80(10)	<0.0001
plasma total cholesterol (mmol/l)	5.1(1.3)	5.3(1.4)	0.43	4.9(1.1)	5.0(1.0)	0.29
plasma HDL cholesterol (mmol/l)	1.2(0.4)	1.0(0.2)	<0.0001	1.6(0.4)	1.2(0.3)	<0.0001
plasma triacylglycerides (mmol/l)	1.70(2.03)	2.32(2.53)	0.047	1.07(0.64)	1.52(0.87)	<0.0001
plasma nonesterified fatty acids (mmol/l)	0.49(0.17)	0.61(0.21)	0.0002	0.63(0.21)	0.72(0.24)	<0.0001
plasma glycerol (μmol/l)	60.0(27.0)	76.8(27.3)	<0.0001	81.9(43.2)	116.8(53.8)	<0.0001
fasting plasma glucose (mmol/l)	5.4(1.2)	6.4(2.3)	0.0006	5.0(0.7)	5.6(1.4)	<0.0001
fasting serum insulin (mIE/l)	7.8(5.3)	20.5(12.0)	<0.0001	6.2(3.4)	14.7(7.9)	<0.0001
HOMA-IR	2.01(1.98)	6.05(4.41)	<0.0001	1.40(0.88)	3.80(2.88)	<0.0001
cell volume (pl)	495(154)	812(193)	<0.0001	475(177)	861(179)	<0.0001
Spontaneous lipolysis (mmol glycerol/2hrs/ESAT)	0.8(0.7)	3.9(2.8)	<0.0001	1.0(0.9)	3.4(2.9)	<0.0001
Stimulated lipolysis (mmol glycerol/2hrs/ESAT)	6.6(5.2)	3.6(1.6)	<0.0001	9.1(6.4)	5.8(7.0)	<0.0001

Where: obese is defined as BMI>30 kg/m^2^; Spontaneous lipolysis rate was calculated as glycerol release divided by the lipid weight of the incubated fat cells; Stimulated lipolysis was calculated as the quotient of glycerol release at the maximum effective isoprenaline concentration divided by the spontaneous rate (no hormone present) of glycerol release from the isolated fat cellscontinuous variables are presented as mean (sd), groups were compared with Student's t-test (unpaired).

SAT gene expression was studied in 114 women, a subset of the cohort. This cohort was previously described [15] and contained the same type of lipolysis data as presented herein. Gene expression of *HIF3A* was measured in 75 subjects from the GENiAL cohort with stored frozen abdominal subcutaneous adipocytes isolated as described below.

### 2.2 Clinical examination

The participants came to the Karolinska University Hospital's clinical research center in the morning after an overnight fast. Height, weight, and waist-to-hip ratio (WHR) were measured. Body fat content was measured via bioimpedance. A venous blood sample was obtained for extraction of DNA and clinical chemistry, which was performed by the hospital's accredited routine clinical chemistry laboratory. HOMA-IR as measure of systemic insulin resistance was calculated from the fasting levels of glucose and insulin as previously described [16]. SAT was obtained via needle aspiration biopsy lateral to the umbilicus as previously described [17]. The estimated abdominal subcutaneous adipose tissue (ESAT) area was calculated using a formula based on WHR, sex, age, waist circumference, and body fat as previously described and validated [18].

### 2.3 Adipose tissue phenotyping

The SAT samples were rapidly rinsed in sodium chloride (9 mg/ml) before removal of visual blood vessels and cell debris and subsequently subjected to collagenase treatment to obtain isolated adipocytes as previously described [19]. Fat cells were incubated as previously described [19]. In brief, cell suspensions (diluted to 2% volume/volume) were incubated for 2 h at 37 °C with air as the gas phase in Krebs–Ringer phosphate buffer (pH 7.4) supplemented with glucose (8.6 mmol/l), ascorbic acid (0.1 mg/ml), and bovine serum albumin (20 mg/ml) either without (spontaneous lipolysis) or with supplementation with synthetic non-selective β-adrenoreceptor agonist isoprenaline (Hässle, Mölndal, Sweden) at increasing concentrations (10^−9^-10^−5^ mol/l; stimulated lipolysis). The amount of glycerol, as a measure of lipolysis, was evaluated in an aliquot of medium at the end of the incubation [20]. This end product of lipolysis, unlike the other final fatty acid metabolites, is not re-utilized by fat cells. The spontaneous lipolysis rate was calculated as the glycerol release to the incubation medium divided by the lipid weight of the incubated fat cells. There was no consensus how to express the lipolysis rates (absolute terms, relative terms, per cell number, or per lipid weight). We expressed isoprenaline-stimulated lipolysis as the quotient of glycerol release at the maximum effective isoprenaline concentration divided by the spontaneous rate (no hormones present) of glycerol release from the isolated fat cells. Spontaneous lipolysis was expressed as glycerol release/cell weight multiplied by the weight of ESAT, that is, an estimate of the total release of glycerol from the ESAT area. The values were log_10_ transformed to improve normality (required for linear regression analysis). These modes of expression were preferred as they in linear regression showed better correlations with clinical parameters in the cohort than other ways of expressing lipolysis (results not shown). Previous *in vitro* studies demonstrated that spontaneous and isoprenaline-stimulated lipolysis were linear for at least 4 h in human subcutaneous fat cells [21].

### 2.4 Genetic analysis

DNA was extracted from whole blood using standard protocols. The samples were genotyped using the UK Biobank Axiom Array r3 platform and called using the Axiom analysis suite (https://assets.thermofisher.com/TFS-Assets/LSG/manuals/axiom_genotyping_solution_analysis_guide.pdf). Samples were excluded for cryptic relatedness, ambiguous sex, or low call rates (<95%). Multi-dimensional scaling, which essentially measures how genetically similar any two individuals are, was conducted in PLINK (http://zzz.bwh.harvard.edu/plink/download.shtml) [22] and the principal components (PCs) calculated for all individuals. PCs 1–4 were plotted and population outliers were excluded by visual inspection. SNPs were excluded for low call rates (<95%), failing Hardy–Weinberg equilibrium (P < 5 × 10^−6^), or low minor allele frequency (MAF < 1%). After quality control, imputation was performed using the haplotype reference consortium panel and, when variants were not available, the 1000G phase 3 reference panel [23]. Post-imputation quality control excluded SNPs with minor allele counts <3 and INFO <0.4 as well as related individuals (one of each pair of first- or second-degree relatives). After quality control, 885 samples and 9,260,588 SNPs were available for phenotypic analysis.

As previously noted, the spontaneous and stimulated lipolysis measurements were log_10_ transformed prior to statistical analysis. A GWAS was conducted in PLINK [22], using linear regression assuming an additive genetic model and adjusting for population structure (PCs1-3), age, sex, and BMI. BMI was adjusted for as the sample size precluded separate analysis in obese and non-obese individuals. Genome-wide significance was set at P < 5 × 10^−8^ and suggestive significance was set at P < 1 × 10^−5^. Only SNPs with MAF >1% were included in the results. SNPs were assigned to loci using the independent-pairwise function in PLINK [22] with default settings. Heritability was calculated using LD score regression (LDSR) [24].

### 2.5 Data mining

To identify SNPs with potentially functional effects on nearby genes, SNPs meeting the threshold for suggestive significance were assessed using the variant effect predictor (VEP) [25] and ANNOVAR [26] software. To identify genotype-specific gene expression patterns or eQTLs, SNPs reaching GWAS significance or those predicted by VEP or ANNOVAR to have functional effects were explored using GTEx [27].

### 2.6 Adipocyte cell culture and transfection with small interfering RNA

Isolation, growth, and differentiation of SAT-derived human mesenchymal stem cells (hMSCs) were previously described [28]. Marker gene expression (Cap analysis gene expression [CAGE] and qPCR data) and lipid accumulation images for these cells were previously published [29].

hMSCs were transfected using a Neon electroporator (Invitrogen, Carlsbad, CA, USA) according to the manufacturer's protocol. Briefly, 1 million of hMSCs at day 1 of differentiation were mixed with 40 nM ON-TARGETplus SMARTpool small interfering RNAs (siRNAs) targeting *HIF3A* or non-targeting siRNA pool (Dharmacon, Lafayette, CO, USA) and electroporated using a 100 μl Neon electroporation tip. Electroporation conditions were 1600 V, 20 ms width, and 1 pulse. Electroporation was repeated until the required amount of cells were collected for the experimental design. Following electroporation, the cells were plated in antibiotic-free medium at a density of 120,000 cells/well in 24-well plates or 12,000 cells/well in 96-well plates and cultured until days 6–13 of differentiation. The medium was collected at days 10 and 13 of differentiation for glycerol measurements as previously described [30]. The amounts of glycerol were normalized to the number of nuclei in each well (see “Quantification of neutral lipids and cell numbers during adipogenesis”).

For *HIF3A* protein analysis, hMSCs were reverse transfected 24 h before induction of adipogenesis using ON-TARGETplus SMARTpool siRNAs targeting *HIF3A* or non-targeting siRNA control #1 (Dharmacon) as previously described [31]. In this experimental design, the RNA and medium were collected at days 2, 6, and 9 after the induction of differentiation. Lipid accumulation was evaluated at day 9. For proteins, cells were collected 72 h post-transfection.

### 2.7 Quantification of neutral lipids and cell numbers during adipogenesis

Lipid accumulation was quantified at differentiation days 10 and 13. hMSCs differentiated *in vitro* were washed with PBS and fixed with 4% paraformaldehyde solution for 10 min at room temperature. The fixed cells were subsequently washed with PBS and stained with BODIPY 493/503 (0.2 μg/mL; Molecular Probes, Thermo Fisher Scientific) and Hoechst 33,342 (2 μg/mL; Molecular Probes) for 20 min at room temperature. Accumulation of intracellular lipids (BODIPY) and cell numbers (Hoechst) were quantified with a CellInsight CX5 High Content Screening (HCS) Platform (Thermo Fischer Scientific) using integrated protocols. Total BODIPY fluorescence (lipid droplets) was normalized to the number of nuclei in each well.

### 2.8 Isolation of RNA and analysis of gene expression

The hMSCs were collected at days 6, 10, and 13 of differentiation for isolation of RNA. Extraction of total RNA, measurement of concentration and purity, and reverse transcription were performed as previously described [30]. Isolation of RNA from frozen abdominal subcutaneous adipocytes was conducted as previously described [15]. Quantitative RT-PCR of the coding genes was performed using commercial TaqMan probes (Applied Biosystems, Foster City, CA, USA). Gene expression was normalized to the internal reference gene 18s. Relative expression was calculated using the 2 (−ΔΔ threshold cycle) method [32].

### 2.9 Analysis of protein expression

The hMSCs were transfected with siRNA against *HIF3A* 24 h prior to the induction of differentiation as previously described. At 72 h post-transfection, the cells were collected to isolate cytosolic and nuclear fractions as previously described [33]. Nuclei obtained from approximately 500,000 cells were analyzed in 150 μL of radioimmunoprecipitation assay buffer (Perce/Thermo Fisher) supplemented with 5 mM of NaF, 1 mM of Na_3_VO_4_, protease inhibitor cocktail set V (Calbiochem), and benzonase (Sigma–Aldrich) on ice for 30 min. Cytosolic proteins were clarified by centrifugation at 14,000 rpm for 30 min. The nuclear and cytosolic proteins were separated by 10% Mini-PROTEAN TGX Stain-Free Precast Gels (Bio-Rad) allowing direct protein visualization in the gels and after transfer onto the membranes. Protein transfer to the PVDF membranes was performed using a Trans-Blot Turbo Transfer System (Bio-Rad). The membranes were blocked in 3% ECL Advance Blocking Agent (GE Healthcare, Little Chalfont, UK). Antibodies against *HIF3A* were obtained from Proteintech (Rosemont, IL, USA) and Prosci (Poway, CA, USA), both used at 1:1000 dilution. Secondary rabbit IgG-horseradish peroxidase antibodies were obtained from Sigma–Aldrich. Antibody antigen complexes were detected by chemiluminescence using an ECL Select Western Blotting Detection Kit (GE Healthcare) in Chemidoc MP (Bio-Rad) and quantified using Image Lab software (Bio-Rad). Total lysate from Hep2D cells (Novus Biologicals, Abingdon, UK) was used as a positive control for *HIF3A* antibodies.

### 2.10 Statistical analysis of clinical and *in vitro* data

The values of clinical variables and results of the *in vitro* experiments are mean ± standard deviation (SD). Lipolysis, glycerol, and triglyceride values were normalized by log_10_ transformation prior to analysis. Standard statistical tests were used including the t-test and single or multiple regression as indicated in the figure/table legends using StatView software (Abacus Concepts Inc, Berkley, CA, USA) or JMP 13 (SAS, Cary, NC, USA).

## 3 Results

Demographic characteristics of all of the participants in the GENiAL cohort are presented in Table 1 and those analyzed in the GWAS are presented in Table 2. The GWAS cohort consisted predominantly of women who were younger and had a higher frequency of obesity than the men. Obesity was associated with higher systemic levels of fasting lipids, glucose, and insulin levels in both genders. Spontaneous lipolysis, measured as the rate of glycerol release from a defined segment of abdominal subcutaneous adipose tissue, was higher in the obese subjects. The stimulated lipolysis, measured as catecholamine-induced divided by basal glycerol release in isolated fat cells, was lower among the obese subjects. As expected, spontaneous and stimulated lipolysis were inversely correlated to one another (R^2^ = 0.49, standardized beta −0.70, p = 1 × 10^−134^).

To assess *in vivo* relevance, spontaneous and stimulated lipolysis measures were assessed for association with systemic metabolic variables (Table 3). Spontaneous lipolysis was positively correlated with the fasting plasma levels of triglycerides, free fatty acids, insulin, and HOMA-IR as measures of systemic insulin resistance after adjusting for age and sex (Table 3). For the triglycerides, insulin, and HOMA-IR, the results remained significant after adjusting to BMI (results not shown).

**Table 3:** Associations between adipocyte spontaneous or stimulated lipolysis and systemic metabolic variables.

	Spontaneous lipolysis		Stimulated lipolysis	
	Standardized beta	P	Standardized beta	P
plasma triglycerides (mmol/l)	0.30	9.0 × 10^−19^	−0.24	3.0 × 10^−14^
plasma nonesterified fatty acids (mmol/l)	0.22	5.3 × 10^−9^	−0.07	0.049
plasma glycerol (μmol/l)	0.22	2.0 × 10^−12^	−0.12	1.0 × 10^−4^
fasting serum insulin (mU/l)	0.57	4.0 × 10^−71^	−0.38	2 × 10^−31^
HOMA_IR_	0.56	3.0 × 10^−67^	−0.38	1.0 × 10^−31^

Spontaneous lipolysis rate was calculated as glycerol release divided by the lipid weight of the incubated fat cells; Stimulated lipolysis was calculated as the quotient of glycerol release at the maximum effective isoprenaline concentration divided by the spontaneous rate (no hormone present) of glycerol release from the isolated fat cells; Analysis was performed using multiple regression adjusting for age, and sex.

### 3.1 GWAS of spontaneous and stimulated lipolysis

**Figure 1:**
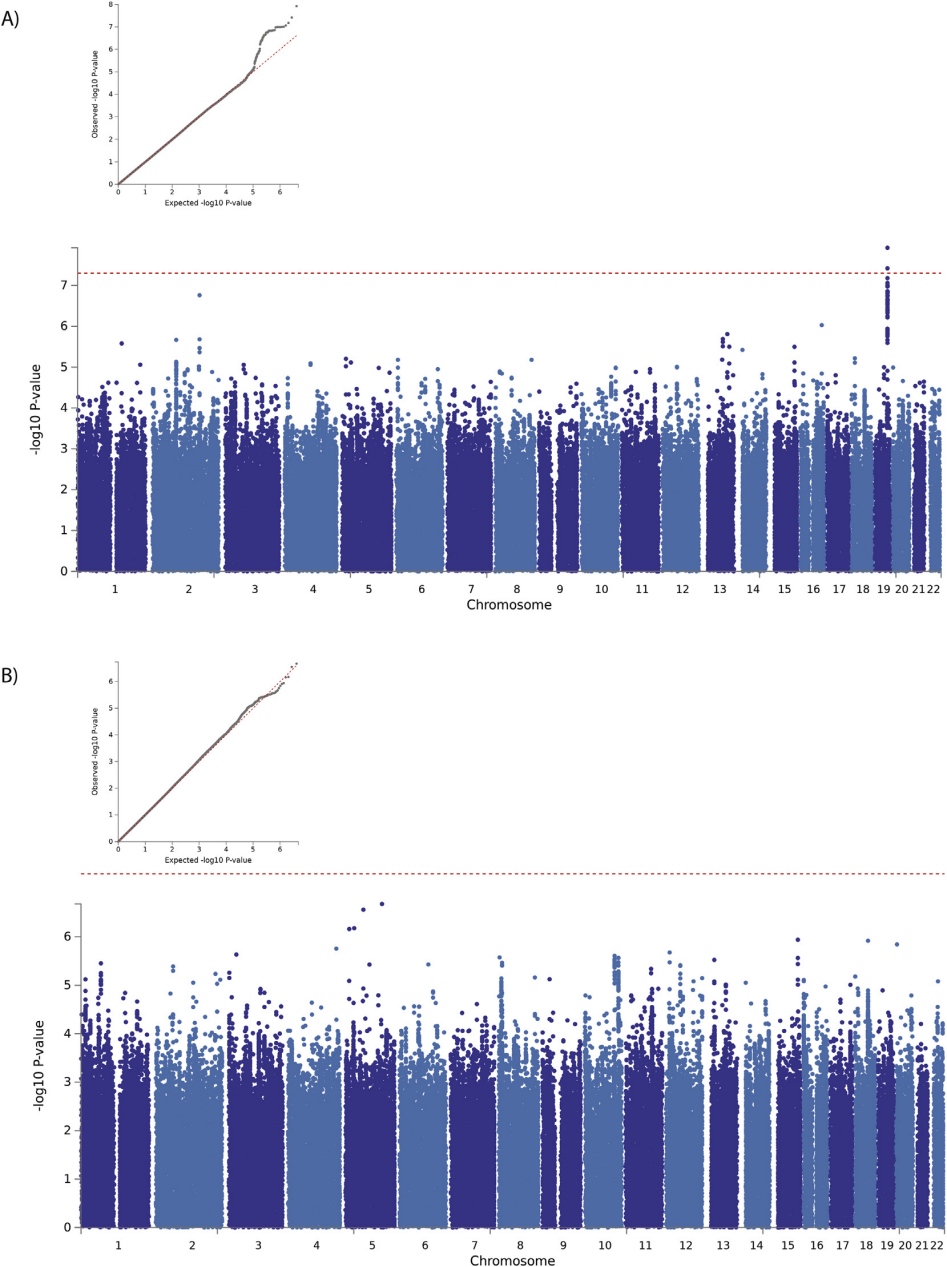
Results of the GWAS of spontaneous and stimulated lipolysis. QQ and Manhattan plots for A) spontaneous lipolysis and B) isoprenaline-stimulated lipolysis. In the QQ plot, the red dotted line indicates the null distribution. In the Manhattan plot, the horizontal red line represents the threshold for GWAS significance (P < 5 × 10^−8^).

**Figure 2:**
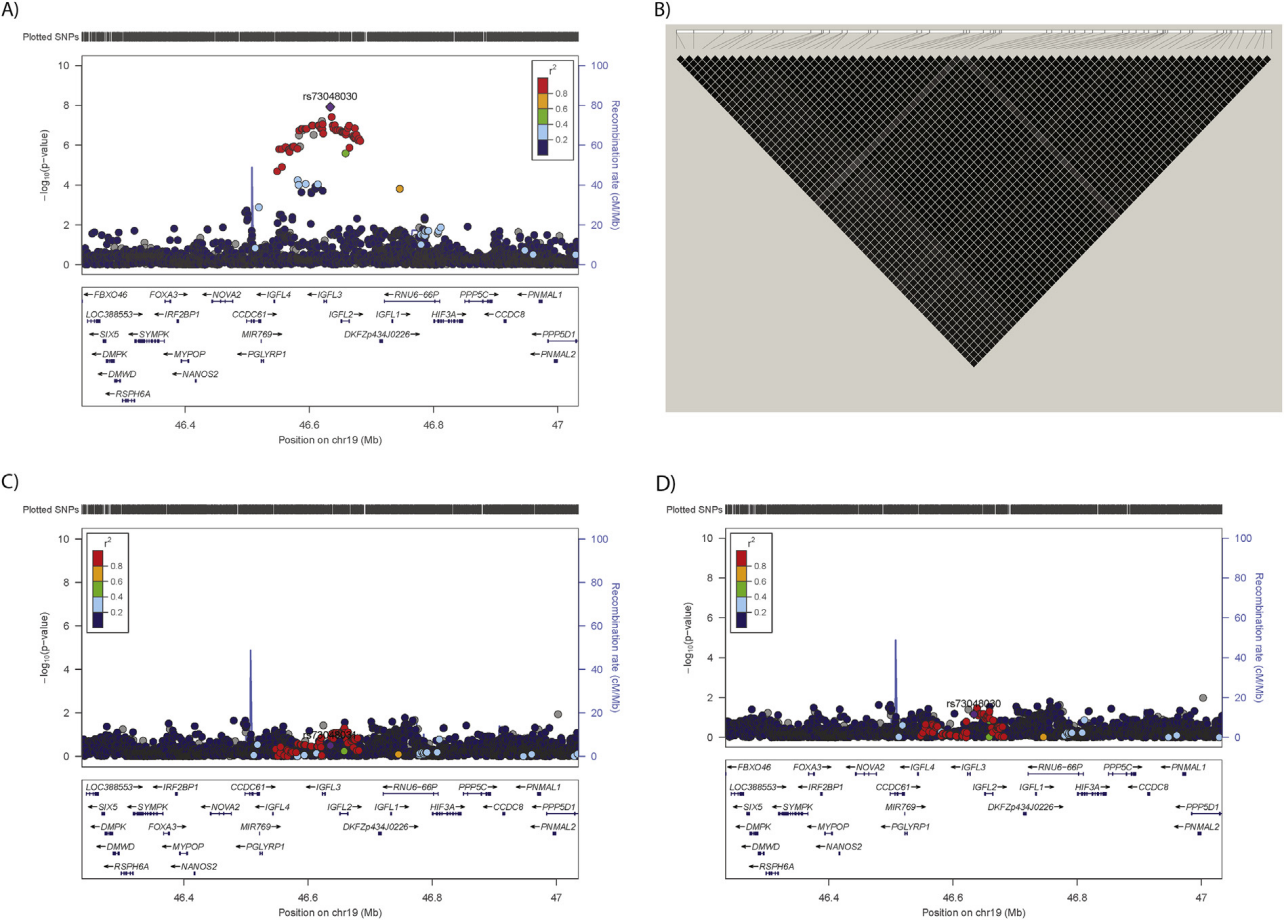
Regional plot of the GWAS-significant spontaneous lipolysis-associated locus on chromosome 19. A) Regional plot of the GWAS-significant locus, B) linkage disequilibrium (LD) of SNPs meeting the threshold for suggestive significance, and C) regional plot after conditional analyses of the GWAS-significant rs73048030 or D) rs73048031. LD colors and values given in R2.

The results are presented in Figure 1 and Supplementary Tables 1 and 2 A total of 112 SNPs in 25 loci demonstrated suggestive associations (P < 1 × 10^−5^) with spontaneous lipolysis (Figure 1A, Table 4, and Supplementary Table 1), including one that demonstrated GWAS-significant (P < 5 × 10^−8^) associated SNPs on chromosome 19. Many genes were potentially impacted by the SNPs in this region of chromosome 19 (Figure 2A). For stimulated lipolysis (Figure 1B), 156 SNPs in 40 loci reached the threshold for suggestive association (Table 4 and Supplementary Table 2); however, no GWAS-significant signals were identified. The sample size precluded analysis stratifying by obesity status. However sensitivity analysis of the GWAS-significant signals demonstrated consistent directions, but weaker effects in the obese vs non-obese individuals (rs73048030: beta = −0.26 Se 0.08 P = 2.40 × 10^−7^ vs beta = −0.55 Se = 0.10 P = 7.46 × 10^−4^, respectively).

**Table 4:** GWAS-significant and suggestive loci for spontaneous lipolysis and suggestive loci for stimulated lipolysis.

						Spontaneous lipolysis						Stimulated lipolysis					
Chr	Pos	SNP	Ref	Alt	Freq	N	Beta	Se	L95	U95	P	N	Beta	Se	L95	U95	P
1	44208924	1:44208924_D_I	I	D	0.85	856	0.144	0.029	0.088	0.200	5.48E-07	874	−0.076	0.022	−0.118	−0.033	0.0006
1	161567916	rs371656041	C	T	0.99	856	0.449	0.095	0.263	0.636	2.64E-06	874	−0.300	0.069	−0.434	−0.165	1.44E-05
1	229572764	rs145251747	D	I	0.98	856	0.429	0.091	0.250	0.608	3.13E-06	874	−0.257	0.070	−0.394	−0.120	0.0003
2	87271929	rs71226525	T	G	0.54	856	0.097	0.020	0.057	0.137	2.16E-06	874	−0.046	0.015	−0.076	−0.016	0.0027
2	170578879	rs369684581	A	G	0.83	856	0.108	0.024	0.061	0.156	9.60E-06	874	−0.056	0.018	−0.092	−0.020	0.0026
2	172907753	rs60231166	T	C	0.74	856	0.121	0.023	0.076	0.166	1.74E-07	874	−0.055	0.017	−0.090	−0.021	0.0016
3	67430871	rs73098354	G	A	0.98	856	−0.266	0.060	−0.383	−0.150	8.80E-06	874	0.192	0.045	0.103	0.280	2.63E-05
4	94856317	rs2918080	G	T	0.78	856	−0.096	0.021	−0.138	−0.054	8.80E-06	874	0.039	0.016	0.007	0.071	0.0164
5	14637275	rs138114267	A	C	0.98	856	0.285	0.063	0.162	0.408	6.27E-06	874	−0.204	0.047	−0.297	−0.111	1.92E-05
5	32955093	rs114601412	T	C	0.95	856	0.176	0.039	0.100	0.253	7.70E-06	874	−0.148	0.030	−0.206	−0.090	6.64E-07
6	6326042	rs9502434	A	G	0.96	856	0.194	0.043	0.110	0.278	6.63E-06	874	−0.129	0.033	−0.192	−0.065	8.72E-05
6	44053018	rs554214639	D	I	0.85	856	−0.111	0.024	−0.158	−0.063	6.38E-06	874	0.065	0.018	0.028	0.101	0.0005
8	133340555	rs111301880	T	C	0.97	856	0.278	0.061	0.158	0.398	6.63E-06	874	−0.206	0.045	−0.295	−0.116	6.90E-06
12	53131676	rs11170254	T	C	0.09	856	−0.141	0.032	−0.203	−0.079	9.83E-06	874	0.112	0.024	0.065	0.160	3.86E-06
13	71658551	rs144654648	T	C	0.99	856	0.389	0.086	0.221	0.556	6.58E-06	874	−0.214	0.065	−0.342	−0.087	0.0010
13	74083355	rs117619390	G	A	0.98	856	−0.293	0.061	−0.413	−0.173	2.05E-06	874	0.152	0.046	0.063	0.242	0.0009
13	90513818	rs9588787	T	C	0.42	856	−0.086	0.018	−0.121	−0.051	1.55E-06	874	0.051	0.014	0.024	0.077	0.0002
13	97727951	rs149528824	G	C	0.99	856	0.417	0.089	0.243	0.591	3.17E-06	874	−0.228	0.068	−0.361	−0.094	0.0008
14	19462974	rs28701881	T	C	0.99	856	0.478	0.103	0.277	0.680	3.80E-06	874	−0.366	0.082	−0.526	−0.205	8.87E-06
15	68691171	rs199600197	I	D	0.11	856	−0.171	0.035	−0.240	−0.102	1.34E-06	874	0.102	0.027	0.050	0.154	0.0001
15	92595793	rs28660218	T	C	0.99	856	0.384	0.082	0.223	0.544	3.19E-06	874	−0.305	0.062	−0.426	−0.183	1.15E-06
16	79960721	rs143486974	A	G	0.98	856	0.360	0.073	0.217	0.503	9.35E-07	874	−0.166	0.056	−0.276	−0.057	0.0030
18	12923043	rs16939974	C	T	0.84	856	0.104	0.023	0.059	0.149	6.08E-06	874	−0.075	0.017	−0.109	−0.041	1.40E-05
19	46633622	rs73048030	A	G	0.98	**856**	**0.377**	**0.066**	**0.249**	**0.506**	**1.20E-08**	874	−0.197	0.049	−0.293	−0.101	5.90E-05
19	46635857	rs73048031	C	T	0.98	**856**	**0.372**	**0.067**	**0.241**	**0.504**	**3.82E-08**	874	−0.197	0.050	−0.294	−0.099	8.74E-05

1	16291470	rs3979178	A	G	0.73	856	0.065	0.019	0.028	0.103	0.0007	874	-0.065	0.014	-0.093	-0.037	7.54E-06
1	72905881	rs7524249	C	A	0.80	856	0.078	0.022	0.035	0.120	0.0003	874	-0.076	0.016	-0.107	-0.044	3.39E-06
2	63587155	rs148533396	T	C	0.98	856	0.245	0.062	0.124	0.366	8.17E-05	874	-0.218	0.047	-0.310	-0.126	4.10E-06
2	87414589	rs540268296	D	I	0.98	856	0.287	0.083	0.124	0.450	0.0006	874	-0.280	0.062	-0.403	-0.158	8.15E-06
2	137306522	rs116525497	G	A	0.98	856	0.249	0.060	0.131	0.367	4.08E-05	874	-0.205	0.046	-0.294	-0.115	8.84E-06
2	218934359	rs55703230	T	C	0.99	856	0.440	0.100	0.243	0.636	1.32E-05	874	-0.347	0.076	-0.496	-0.198	5.82E-06
2	236618480	rs560405754	A	C	0.99	856	-0.282	0.094	-0.466	-0.098	0.0028	874	0.314	0.070	0.177	0.450	7.71E-06
3	2428151	rs1032784	A	G	0.75	856	-0.078	0.020	-0.118	-0.038	0.0001	874	0.070	0.015	0.040	0.100	5.51E-06
3	28133397	rs1506686	A	C	0.99	856	0.321	0.087	0.150	0.492	0.0003	874	-0.314	0.066	-0.443	-0.184	2.32E-06
3	116686382	rs590225	T	C	0.82	856	0.078	0.023	0.033	0.124	0.0008	874	-0.078	0.017	-0.112	-0.044	8.21E-06
4	177130984	rs73007116	G	T	0.91	856	0.105	0.033	0.040	0.171	0.0016	874	-0.118	0.025	-0.166	-0.070	1.75E-06
5	14799937	rs114867704	T	C	0.97	856	0.246	0.060	0.128	0.363	4.69E-05	874	-0.222	0.044	-0.309	-0.135	6.91E-07
5	66547938	5:66547938_G_A	A	G	0.96	856	0.157	0.049	0.061	0.253	0.0015	874	-0.190	0.037	-0.262	-0.118	2.76E-07
5	88866702	rs74574939	G	T	0.99	856	0.250	0.076	0.101	0.398	0.0010	874	-0.267	0.057	-0.379	-0.154	3.74E-06
5	134881956	rs72787161	T	G	0.98	856	0.328	0.074	0.183	0.474	1.04E-05	874	-0.288	0.055	-0.395	-0.180	2.10E-07
6	105831370	rs117269303	T	C	0.99	856	0.270	0.076	0.121	0.420	0.0004	874	-0.263	0.056	-0.373	-0.152	3.73E-06
6	123139459	rs35141697	D	I	0.75	856	-0.067	0.020	-0.107	-0.028	0.0009	874	0.070	0.015	0.040	0.100	4.94E-06
7	41024130	rs200674393	I	D	0.98	856	-0.329	0.077	-0.481	-0.177	2.45E-05	874	0.305	0.059	0.191	0.420	2.29E-07
8	3902830	rs79055631	T	G	0.95	856	0.138	0.042	0.057	0.220	0.0009	874	-0.148	0.031	-0.209	-0.086	2.66E-06
8	10375973	rs111818085	I	D	0.89	856	0.064	0.027	0.012	0.117	0.0163	874	-0.094	0.020	-0.133	-0.055	2.78E-06
8	13095207	rs7007799	G	A	0.87	856	0.094	0.027	0.041	0.146	0.0005	874	-0.094	0.020	-0.133	-0.054	3.45E-06
9	26337144	rs10967318	A	G	0.99	856	0.311	0.101	0.112	0.509	0.0023	874	-0.321	0.071	-0.460	-0.181	7.50E-06
10	109418980	rs1902740	T	C	0.28	856	0.082	0.019	0.045	0.119	1.49E-05	874	-0.069	0.014	-0.097	-0.042	1.21E-06
11	94458034	rs117762377	A	G	0.99	856	-0.174	0.079	-0.328	-0.019	0.0277	874	0.262	0.057	0.150	0.373	4.58E-06
12	14094162	rs148192549	C	T	0.99	856	0.238	0.087	0.068	0.408	0.0062	874	-0.304	0.064	-0.429	-0.179	2.11E-06
12	101524071	12:101524071_G_A	A	G	0.98	856	0.158	0.064	0.032	0.285	0.0143	874	-0.209	0.047	-0.300	-0.117	8.29E-06
12	133262001	rs11147005	T	C	0.32	856	0.045	0.019	0.009	0.082	0.0148	874	-0.062	0.014	-0.089	-0.035	7.14E-06
13	30567601	rs142268497	C	T	0.99	856	-0.246	0.085	-0.413	-0.080	0.0038	874	0.301	0.064	0.176	0.427	3.00E-06
13	73025966	rs9318075	A	G	0.64	856	0.051	0.018	0.016	0.086	0.0041	874	-0.059	0.013	-0.085	-0.033	9.92E-06
16	1595600	rs56342298	T	C	0.95	856	0.108	0.039	0.031	0.185	0.0064	874	-0.132	0.029	-0.189	-0.074	7.79E-06
17	73299631	rs9892812	G	A	0.47	856	-0.044	0.018	-0.079	-0.009	0.0146	874	0.059	0.013	0.033	0.085	9.79E-06
18	1772503	rs541861443	A	T	0.42	856	-0.078	0.019	-0.116	-0.041	4.75E-05	874	0.065	0.014	0.037	0.094	6.62E-06
18	48657387	18:48657387_C_T	T	C	0.73	856	0.084	0.020	0.045	0.124	3.66E-05	874	-0.074	0.015	-0.104	-0.045	1.21E-06
20	4213175	rs79601367	A	G	0.96	856	0.160	0.045	0.071	0.249	0.0005	874	-0.167	0.034	-0.234	-0.099	1.44E-06
22	32335786	rs138027893	A	C	0.95	856	0.131	0.040	0.052	0.210	0.0012	874	-0.135	0.030	-0.194	-0.076	8.31E-06

Beta, effect of Alt allele; Freq, frequency of Alt allele; Spontaneous lipolysis rate was calculated as glycerol release divided by the lipid weight of the incubated fat cells multiplied by the weight of ESAT; Stimulated lipolysis was calculated as the quotient of glycerol release at the maximum effective isoprenaline concentration divided by the spontaneous rate (no hormone present) of glycerol release from the isolated fat cells. Where SNPs were suggestive for both traits, they are listed under spontaneous lipolysis. The most significant SNP in each locus i listed in the table.

There was a strong overlap between SNPs associated with spontaneous and stimulated lipolysis; all of the SNPs displaying suggestive associations with spontaneous lipolysis were nominally associated with stimulated lipolysis (Supplementary Table 1), whereas 149 of the 156 SNPs displaying suggestive associations with stimulated lipolysis were nominally associated with spontaneous lipolysis (Supplementary Table 2). This was expected as spontaneous and stimulated lipolysis are correlated (inversely) and share several regulatory steps in signaling to the lipases that ultimately hydrolyze triglycerides [[1], [2], [3]].

Using LDSR, SNP heritability (observed scale) was estimated as 14.2% (h^2^_SNP_ = 0.1426; SE = 0.468) for spontaneous lipolysis and 26.4% (h^2^_SNP_ = 0.264; SE = 0.454) for stimulated lipolysis. Of note, the summary statistics meta-data for both spontaneous and stimulated lipolysis suggested that the files were not suitable for genetic correlation analyses (the mean chi-square was too low). Given that the sample size was <1,000 individuals, this was unsurprising.

### 3.2 Chromosome 19 locus was significantly associated with spontaneous lipolysis

The genome-wide significant spontaneous lipolysis-associated chromosome 19 locus (Figure 2A) encompasses a region of ∼130 Kb that includes two GWAS-significant SNPs and 68 suggestive SNPs (Supplementary Table 3). The linkage disequilibrium (LD) between SNPs meeting the threshold for suggestive evidence of association (Figure 2B) and the non-significant association of these SNPs with spontaneous lipolysis analyses when conditioning on the GWAS-significant SNPs, rs73048030 (Figure 2C), or rs73048031 (Figure 2D) demonstrated that this locus contained only one association signal (Supplementary Table 3). All of the SNPs in the chromosome 19 locus demonstrating GWAS-significant evidence of association with spontaneous lipolysis were assessed for potential functional effects using VEP and ANNOVAR. None of the SNPs were predicted to have significant or deleterious effects on nearby genes. The most severe consequence for each SNP is reported in Supplemental Table 4. The chromosome 19 locus was previously associated with inflammatory and hematological traits (Supplementary Table 5) [[34], [35], [36], [37], [38], [39], [40]]. This locus was also previously associated with lipoprotein disorders in UK Biobank (albeit in a case–control analysis with only 51 cases, http://pheweb.sph.umich.edu/pheno/277.51).

### 3.3 Expression analysis for chromosome 19 locus candidate gene identification

**Figure 3:**
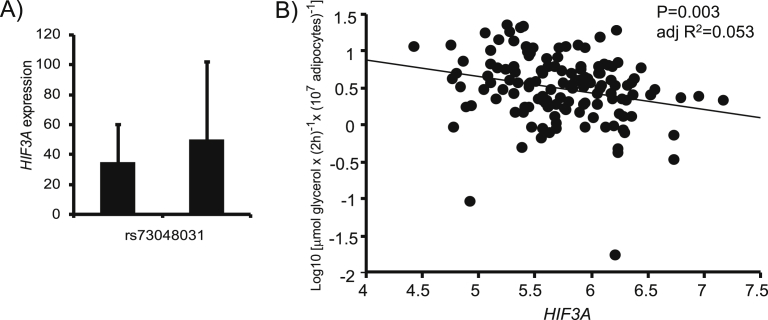
Functional validation of *HIF3A* function in human adipocytes. A) HIF3A gene expression during *hMSC* differentiation *in vitro*. Data retrieved from FANTOM5 dataset (http://fantom.gsc.riken.jp/5/). B) and D-E) Expression of *HIF3A* was knocked down using siRNA in hMSCs 24 h after induction of differentiation and followed until differentiation days 6, 10, and 13, upon which B) the expression of *HIF3A, LIPE, PLIN1*, and *PNPLA2* was monitored, D) glycerol amount in medium was measured, E) accumulation of neutral lipids was evaluated, and F) the expression of ADIPOQ and FABP4 was measured. C) The expression of *HIF3A* was knocked down using siRNA in hMSCs 24 h before induction of adipogenic differentiation of hMSCs; 72 h post-transfection, the cells were collected, fractionated to the cytosolic and nuclear fraction, and the proteins were analyzed by Western blotting. The expression of *HIF3A* was normalized to the total protein amount. Results in B-E are based on four biological/independent experiments. The expression of genes was normalized to the reference gene 18s. The results were analyzed using t-tests and presented as fold change ± SD relative to negative control of a corresponding time point (Neg C). Results in C) were analyzed using one-sided t-tests and presented as fold change ± SD relative to negative control (Neg C). ∗∗∗P < 0.005, ∗∗P < 0.01, ∗P < 0.05.

All of the transcripts in the chromosome 19 region bordered by a recombination rate of 25% from the lead SNPs were examined for expression in publicly available transcriptome data of our human adipocyte cultures and in isolated mature abdominal adipocytes from our clinical samples available in the FANTOM5 dataset (http://fantom.gsc.riken.jp/5/) and [29]. Only *HIF3A* was expressed in the isolated mature adipocytes (Supplementary Table 6) and precursor cells, that is the hMSCs undergoing differentiation to adipocytes (Figure 3A). *PPP5C* was expressed in the mature adipocytes but not precursors, so was not included in further analysis.

**Figure 4:**
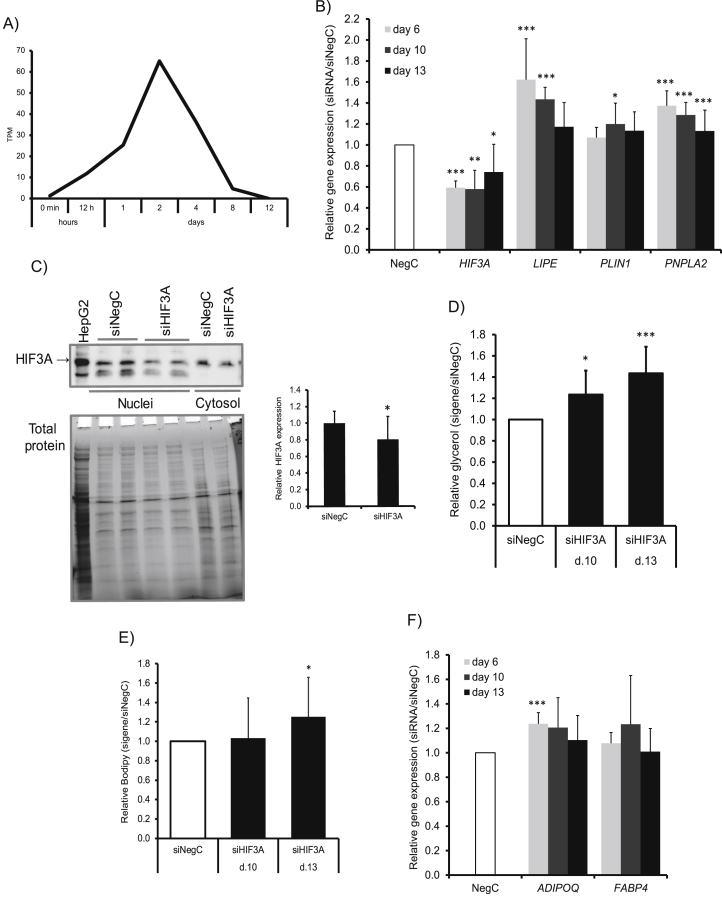
A) Genotype-specific gene expression for *HIF3A*, with higher levels observed with 2 copies of rs73048031-C. Gene expression expressed relative to 18 S. Genotype groups were compared with Student's t-test. P = 0.076 (two-sided), P = 0.038 (one-sided). B) Relationship between abdominal SAT *HIF3A* expression and spontaneous lipolysis.

We also examined the genotype-dependent gene expression in the chromosome 19 locus. According to GTEx [27], the genome-wide significant SNPs on chromosome 19, rs73048030, and rs73048031 were eQTLs for *IGL3* in the skin, but not for *HIF3A* in any investigated organ including the white adipose tissue (Supplementary Table 4). Adipose tissue comprises different cell types that can mask an eQTL effect on a single cell type. To test an adipocyte-specific effect of rs73048030 and rs73048031, we measured *HIF3A* in the isolated frozen adipocytes from GENiAL (N = 75). The common allele of rs73048031-C was marginally associated with higher gene expression (P = 0.076 two-sided; P = 0.038 one-sided) (Figure 4A). Due to the low MAF of rs73048031, no homozygous subject for the rare allele was included in the eQTL analysis. The results did not remain significant if adjusted for age, sex, and BMI, which was not surprising given the limited size of our cohort. The results for rs73048030 were similar although non-significant due to the lower genotype call rate (results not shown). No genotype-specific expression patterns were identified for *PPP5C*.

In addition, *HIF3A* in abdominal SAT in relation to lipolysis was assessed in a subset of the study cohort with lipolysis and gene expression measurements (114 women with a wide variation in BMI). *HIF3A* expression was inversely correlated with spontaneous lipolysis (Figure 4B). Based on these findings, *HIF3A* was further functionally evaluated.

### 3.4 Effect of candidate gene manipulation on adipose tissue biology

The expression of *HIF3A* during differentiation of hMSCs increased at the very beginning of differentiation, reaching peak expression at day 2 of differentiation and decreasing thereafter to almost the same level as in the beginning of differentiation (Figure 3A). Based on the high expression early in differentiation, we knocked down *HIF3A* using siRNA in the hMSCs at day 1 and followed the cells to differentiation days 10 and 13. This resulted in 45–50% decreased *HIF3A* expression at days 5, 9, and 12 post-transfection (that is, days 6, 10, and 13 of differentiation, Figure 3B); thus, approximately 50% of knockdown was left by the end of differentiation. In contrast, the expression levels of the genes central to lipolysis (hormone-sensitive lipase (*LIPE* ), perilipin 1 (*PLIN1*), and adipose triglyceride lipase (*PNPLA2*)) increased by *HIF3A* knockdown. The expression of *LIPE* increased by at least 60-40% at days 6 and 10. The expression of *PNPLA2* increased by 40, 30, and 15%, respectively, at days 6, 10, and 13. The expression of *PLIN1* increased by ∼20% at day 10 only (Figure 3B). The effect of gene silencing naturally gradually diminished due to the long post-transfection time of almost two weeks. We analyzed the effects of siRNA on *HIF3A* protein at day 2 of differentiation due to its highest expression at the beginning of differentiation. We detected a small but significant downregulation of *HIF3A* protein in the nuclear fraction but not in the cytosolic fraction (Figure 3C).

We further evaluated the glycerol levels in the conditioned medium as a marker of lipolysis. The glycerol levels increased significantly by approximately 20-50% at days 10 and 13, respectively (Figure 3D). We also evaluated the effects on lipid accumulation of *HIF3A* knockdown. The amount of neutral lipids increased by 30% at day 13 but not day 10 of differentiation (Figure 3E). Finally, *HIF3A* knockdown had only minor effects on adipocyte-specific genes. The expression of *ADIPOQ* increased by 25% at day 6 while the expression of *FABP4* was not significantly affected (Figure 3F).

### 3.5 Analysis of previous clinically relevant associations for lipolysis-associated genetic loci

We next assessed whether lipolysis-associated SNPs in the present study were associated with obesesity-related clinical traits. None of the suggestive lipolysis-associated SNPs were previously reported to be associated with obesity (BMI, WHR, and % body fat) or related metabolic traits (type 2 diabetes, insulin resistance, and plasma lipids) according to the GWAS catalogue (https://www.ebi.ac.uk/gwas/, 20190410). However, a few SNPs were located in genes or within +/−100 Kbps of SNPs reported to be associated with these traits, for example, a locus on chromosome 2 harboring *METAP1D* was found to be associated with % body fat [41] and loci on chromosome 15 (*SLCO3A1*) and 18 with BMI [8,42] (Supplementary Table 7).

### 3.6 Data mining of spontaneous or stimulated lipolysis loci

The SNPs displaying suggestive associations with spontaneous or stimulated lipolysis were next investigated for genotype-specific gene expression (or eQTLs). Among the SNPs represented in GTEx, the SNPs associated with spontaneous lipolysis also demonstrated genotype-specific expression patterns on chromosome 2 for *PLGLB1* in the SAT as well as on chromosome 19 for *PPP5D1* in the brain and *IGFL3* in the skin (Supplementary Table 4 and Supplementary Table 8). SNPs in six loci associated with stimulated lipolysis demonstrated eQTLs. The most interesting was rs9892812 on chromosome 17, which influenced gene expression in the SAT of *NUP85*, *GGA3*, and *MRPS7* (Supplementary Table 4 and Supplementary Table 8). Other notable findings include rs11147005 on chromosome 12, the *P2RX2* levels in the visceral adipose tissue, and many SNPs on chromosome 10 influencing *ATE1* levels in the skeletal muscle (Supplementary Table 4 and Supplementary Table 8). Brief descriptions of genes highlighted by these analyses are presented in Supplementary Table 9. None of the eQTL-associated genes have established roles in lipolysis regulation. There are many caveats to consider when interpreting eQTL data (for example, the sample size, data collection time and method, and tissue selection); however, these data are included to be as comprehensive as possible.

## 4 Discussion

In this first GWAS of human fat cell lipolysis, we identified one genetic locus significantly associated with spontaneous lipolysis and multiple additional loci with suggestive associations with spontaneous and stimulated lipolysis. Furthermore, we provided evidence of *HIF3A* as a candidate gene with a key role in regulating the expression of genes central to lipolysis.

Regulation of SAT lipolysis is not well understood. Prior genetic studies implicated coding variants in or near genes encoding proteins with established and specific roles in lipolysis and glycerol release [43]. In contrast, in this unique cohort, we identified the SNPs associated with lipolysis that are not located in or near genes encoding proteins with established roles in lipolysis. This is in line with studies of other complex traits in which a GWAS of a well-defined phenotype can highlight novel biology. That prior studies were not validated herein suggests that they may represent spurious associations due to underpowered study designs.

While several nominal loci were identified, for robustness, we focused on the genome-wide significant locus on chromosome 19. This is a gene-dense region with high LD. Among the genes in this locus, only *HIF3A* was expressed in mature and precursor adipocytes and constituted an eQTL and was thus the focus of further evaluation. We provided functional support for transcription factor *HIF3A* explaining the association between the chromosome 19 locus and lipolysis: knockdown of *HIF3A* in hMSCs increased glycerol release and, consistent with this, increased the expression of genes encoding the major lipolysis-regulating lipases, that is, *LIPE* and *PLNPLA2.* Furthermore, the major lipid droplet-coating protein in fat cells, *PLIN1,* which is also critical for lipolysis, was influenced by *HIF3A* knockdown. In agreement with an inhibitory effect of *HIF3A* on lipolysis, we observed an inverse correlation between *HIF3A* expression and SAT spontaneous lipolysis in women and borderline nominally significant (P < 0.05) association with the rs73048031 genotype, whereby the spontaneous lipolysis-decreasing allele (C) was associated with increased *HIF3A* gene expression levels. We were unable to exclude the possibility that genes encoding secreted proteins could control lipolysis through distant-acting systemic effects, for example, *IGFL3*, which is primarily expressed in the skin and comprises a cis-eQTL in this organ for the lipolysis-associated SNPs on chromosome 19. However, there was no evidence that *IGFL3* was present in the general circulation, making this scenario less likely.

The lipolysis-associated SNPs on chromosome 19 were not previously reported to be associated with clinical traits involving altered lipolysis, for example, adiposity, insulin resistance, or dyslipidemia, according to the GWAS catalogue. Furthermore, mice with a targeted disruption of the *HIF3A* locus displayed cardiac and pulmonary remodeling [44], but to the best of our knowledge, no phenotypes related to altered lipid or glucose metabolism have been reported. However, the *HIF3A* locus more generally has previously been associated with lipoprotein subclasses in Finns [45], which might be related to dysfunctional lipolysis. Furthermore, the influence of *HIF3A* on lipolysis could go beyond the genetic effects. In addition to hypoxia, *HIF3A* expression has also been reported to be regulated by the anti-lipolytic hormone insulin [46]. Furthermore, SAT CpG methylation of *HIF3A* has been associated with gene expression and BMI [47]. Also, reported results supported the hypothesis that *HIF3A* methylation is secondary to, rather than causal of, obesity [48]. We cannot exclude the possibility that the SNPs in the lipolysis-associated locus influenced DNA methylation. *HIF3A* could possibly represent a mechanistic link between obesity and metabolic complications since SAT gene expression is inversely correlated with systemic insulin resistance [49].

Hypoxia inducible factors (HIFs) are heterodimeric transcription factors that regulate adaptive responses to low oxygen tension [50]. Ectopic expression of *HIF3A* has been shown to enhance the adipogenic potential in mouse fat cell line 3T3-L1 cells [51]. In this study, knockdown of *HIF3A* did not suppress markers of adipocytes (*FABP4* and *ADIPOQ*) and increased lipid accumulation, suggesting that the effects are species dependent. *HIF3A* binding motifs are not sufficiently well described to determine via bioinformatics analyses whether HIF3A directly represses *LIPE*, *PNPLA2*, and *PLIN1*. Experiments to address this were beyond the scope of the present study.

How regulation of lipolysis is related to traits such as obesity and insulin resistance is an area of obvious interest. However, the many loci influencing metabolic traits likely act through a variety of different mechanisms, with only a handful acting through lipolysis. Therefore, the gold standard methods (genetic correlation and polygenic risk scores) that consider the whole genome are inappropriate and could be misleading. Consideration of specific loci could provide more accurate estimates; however, defining the choice of loci is complex. For example, only the loci with evidence of genotype-specific gene expression patterns in adipose tissue could be considered, or with supportive experimental data, but these methods are fraught with issues. Expression data are limited in sample size, collection time points, and tissue selection, to name only a few concerns. Similarly, only a fraction of loci has been thoroughly studied experimentally. While this information provides additional evidence, there is a significant amount of bias that should be considered in their interpretation (while the observed associations could be true, lack of associations cannot be taken as true findings).

To date, lipolysis has not been extensively studied, and consensus in methodology is lacking. The lipolysis measurements used in this study were those most reflective of (best correlations with) clinical parameters. The limitations of this study include lack of replication, as we are unaware of any other datasets with comparable phenotypes of sufficiently large size to provide reasonable power for replication. The size of the cohort might be the reason for only finding one genome-wide significant locus for the lipolysis phenotypes. Nevertheless, the validity of our findings is supported by first, consistent effects of suggestive findings on the two phenotypes. That is, the effects on spontaneous lipolysis were consistently the inverse of the effects on stimulated lipolysis. Second, many SNPs showed effects on gene expression in adult adipose tissues. Finally, manipulation of the candidate gene expression levels in the GWAS-significant locus demonstrated clear effects on lipolysis. *HIF3A* has multiple splice variants, the expression of which are epigenetically regulated [52]. Whether there are also transcript-specific genetic effects on *HIF3A* expression remains to be determined. Finally, this study was underpowered for sex-specific analysis.

## 5 Conclusions

In conclusion, this first GWAS of adipocyte lipolysis identified a locus on chromosome 19 that was significantly associated with spontaneous lipolysis, demonstrated that genetic regulation of spontaneous and stimulated lipolysis overlaps, and provided evidence for *HIF3A* as a strong candidate gene with a novel and key regulatory function in SAT lipolysis in humans. We also highlighted multiple additional genetic loci with suggestive associations with lipolysis, including some that were previously linked to body fat storage and distribution.

## 6 Data availability

Summary statistics for the spontaneous and stimulated lipolysis GWAS are available upon request.
