# Corrigendum to “Genome-wide association study of adipocyte lipolysis in the GENetics of adipocyte lipolysis (GENiAL) cohort” [Molecular Metabolism 34 (2020) 85–96]

**DOI:** 10.1016/j.molmet.2020.101072

**Published:** 2020-09-08

**Authors:** Agné Kulyté, Veroniqa Lundbäck, Cecilia M. Lindgren, Jian'an Luan, Luca A. Lotta, Claudia Langenberg, Peter Arner, Rona J. Strawbridge, Ingrid Dahlman

**Affiliations:** 1 Lipid laboratory, Department of Medicine Huddinge, Karolinska Institute, Stockholm, Sweden; 2 Big Data Institute at the Li Ka Shing Center for Health Information and Discovery, University of Oxford, Oxford, UK; 3 Wellcome Center for Human Genetics, Nuffield Department of Medicine, University of Oxford, Oxford, UK; 4 National Institute for Health Research Oxford Biomedical Research Center, Oxford University Hospitals NHS Foundation Trust, John Radcliffe Hospital, Oxford, UK; 5 Program in Medical and Population Genetics, Broad Institute, Cambridge, MA, USA; 6 MRC Epidemiology Unit, University of Cambridge, Cambridge, UK; 7 Institute of Health and Wellbeing, University of Glasgow, Glasgow, UK; 8 Department of Medicine Solna, Karolinska Institute, Stockholm, Sweden; 9 Health Data Research UK, UK

The authors regret that original paper was published with errors in Table 2. The number of men have been switched. Correct values are

Men

Nonobese n = 163,

Obese n = 89.

The authors apologies for any inconvenience caused.

