# Supplementary figures and images for: Genome-wide association study of adipocyte lipolysis in the GENetics of adipocyte lipolysis (GENiAL) cohort
Source: Mol Metab. 2020 Jan 25;34:85–96. doi: 10.1016/j.molmet.2020.01.009 (PMC7021539; doi:10.1016/j.molmet.2020.01.009)

Supplementary Figure 1


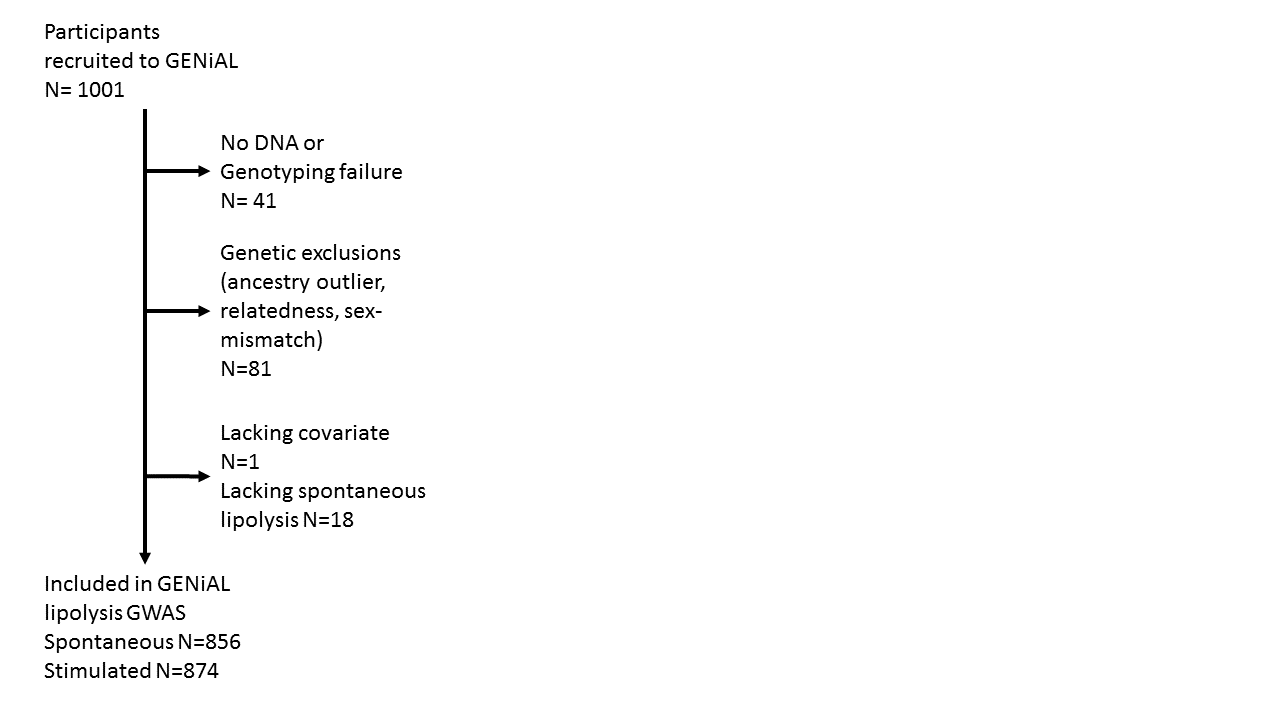

Supplement: Multimedia component 10 [file mmc10.docx]
